# Supplementary material for: The interactome of intact mitochondria by cross-linking mass spectrometry provides evidence for coexisting respiratory supercomplexes
Source: Mol Cell Proteomics. 2017 Dec 8;17(2):216–32. doi: 10.1074/mcp.RA117.000470 (PMC5795388; doi:10.1074/mcp.RA117.000470)
Supplement: Supplemental Data [file supp_RA117.000470_133922_0_supp_23978_fzffwf.zip › spectra_annotation/mito_spectra_annotation/425-1-5-1-6-1.pdf]

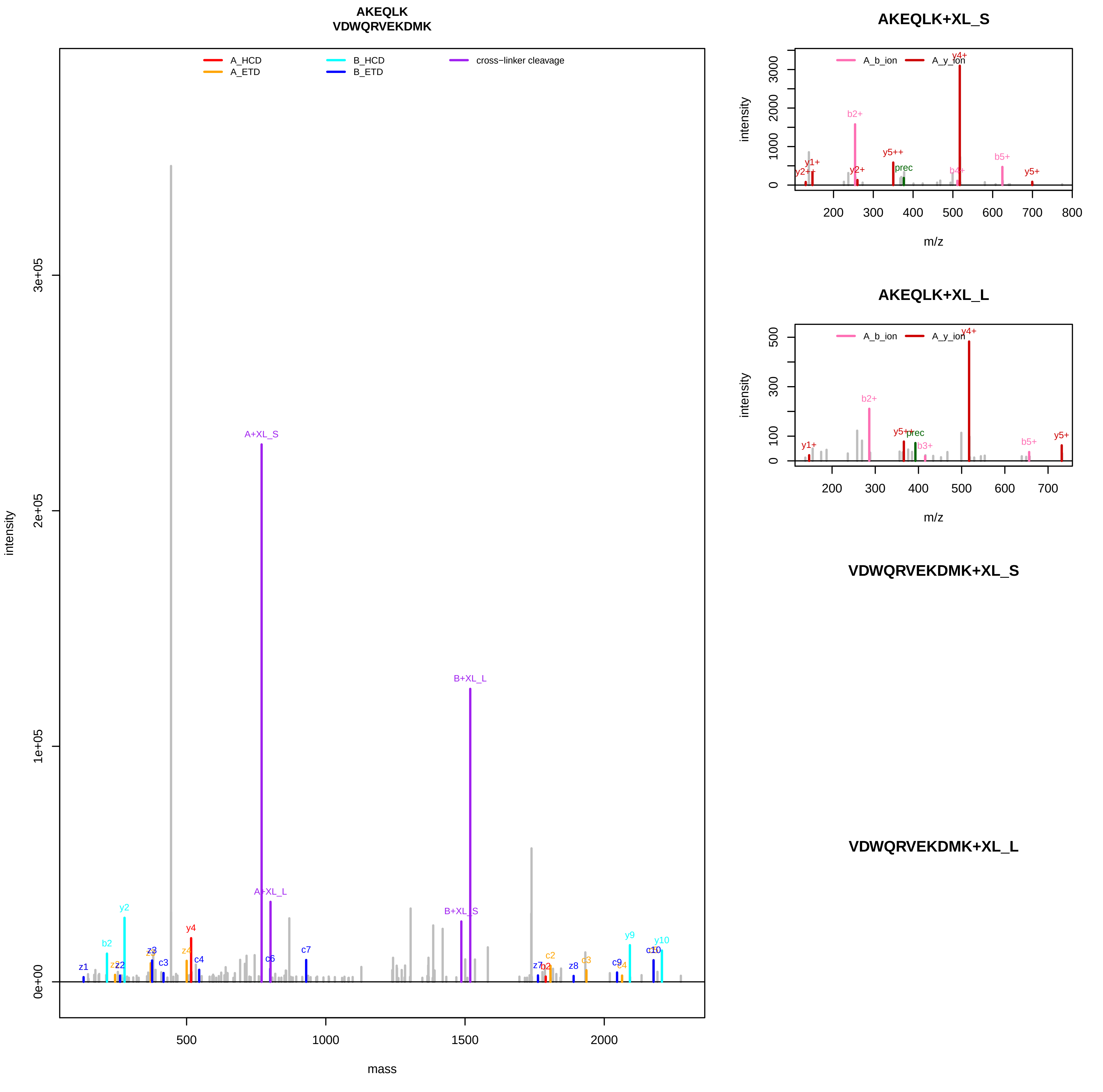
AKEQLK
VDWQRVEKDMK
A_HCD
A_ETD
B_HCD
B_ETD
cross−linker cleavage
intensity
mass
0e+00
1e+05
2e+05
3e+05
500
1000
1500
2000
A+XL_S
A+XL_L
B+XL_S
B+XL_L
z1
b2
z2
y2
z3
c3
z4
y4
c4
c6
c7
z7
b2
c2
z8
c3
c9
c4
c10
y9
y10
AKEQLK+XL_S
A_b_ion
A_y_ion
intensity
m/z
0
1000
2000
3000
200
300
400
500
600
700
800
y2++
y1+
b2+
y2+
y5++
prec
b4+
y4+
b5+
y5+
AKEQLK+XL_L
A_b_ion
A_y_ion
intensity
m/z
0
100
300
500
200
300
400
500
600
700
y1+
b2+
y5++
prec
b3+
y4+
b5+
y5+
VDWQRVEKDMK+XL_S
VDWQRVEKDMK+XL_L
